# A bibliometric analysis on tobacco regulation investigators

**DOI:** 10.1186/s13040-015-0043-7

**Published:** 2015-03-21

**Authors:** Dingcheng Li, Janet Okamoto, Hongfang Liu, Scott Leischow

**Affiliations:** 1 Department of Biomedical Statistics and Informatics, Mayo Clinic, Rochester, MN USA; 2 Department of Hemotology/Oncology Mayo Clinic, Scottsdale, AZ Arizona

**Keywords:** Author topic modeling, Bibliometric analysis, Tobacco regulation science, FDA, Principle investigators

## Abstract

**Background:**

To facilitate the implementation of the Family Smoking Prevention and Tobacco Control Act of 2009, the Federal Drug Agency (FDA) Center for Tobacco Products (CTP) has identified research priorities under the umbrella of tobacco regulatory science (TRS). As a newly integrated field, the current boundaries and landscape of TRS research are in need of definition. In this work, we conducted a bibliometric study of TRS research by applying author topic modeling (ATM) on MEDLINE citations published by currently-funded TRS principle investigators (PIs).

**Results:**

We compared topics generated with ATM on dataset collected with TRS PIs and topics generated with ATM on dataset collected with a TRS keyword list. It is found that all those topics show a good alignment with FDA’s funding protocols. More interestingly, we can see clear interactive relationships among PIs and between PIs and topics. Based on those interactions, we can discover how diverse each PI is, how productive they are, which topics are more popular and what main components each topic involves. Temporal trend analysis of key words shows the significant evaluation in four prime TRS areas.

**Conclusions:**

The results show that ATM can efficiently group articles into discriminative categories without any supervision. This indicates that we may incorporate ATM into author identification systems to infer the identity of an author of articles using topics generated by the model. It can also be useful to grantees and funding administrators in suggesting potential collaborators or identifying those that share common research interests for data harmonization or other purposes. The incorporation of temporal analysis can be employed to assess the change over time in TRS as new projects are funded and the extent to which new research reflects the funding priorities of the FDA.

**Electronic supplementary material:**

The online version of this article (doi:10.1186/s13040-015-0043-7) contains supplementary material, which is available to authorized users.

## Background

To facilitate the implementation of the Family Smoking Prevention and Tobacco Control Act (FSPTCA) of 2009, the Federal Drug Agency (FDA) Center for Tobacco Products (CTP) was formed to oversee tobacco regulatory activities. Its responsibilities include setting performance standards, reviewing premarket applications for new and modified risk tobacco products, requiring new warning labels, and establishing and enforcing advertising and promotion restrictions. In order to meet these responsibilities, the CTP has identified research priories for tobacco regulatory science (TRS) in order to inform and guide the CTP’s regulatory decision-making. While tobacco researchers have been examining some of the CTP’s TRS research priorities for many years, they have not necessarily been doing so under the umbrella or specific title of ‘tobacco regulatory science’. Therefore, examining and identifying research topics from the corpus of TRS work could help to more clearly define this growing research area. In this paper, we applied author topic modeling (ATM) [1], a variation of Latent Dirichlet Allocation (LDA) [2], to simultaneously model the content of documents and the interests of authors. Namely, given the broader TRS research field, we attempted to discover topics as well as general research interests utilizing MEDLINE citations for currently funded TRS investigators.

LDA is known for its ability to model document contents as a mixture of topics (which comprise words describing similar things). This results in improvements in the study of hidden semantics of documents compared with previous models like Latent Semantic Indexing (LSI) [3], probabilistic LSI [4], vector semantics [5] and so on. Modeling interests of authors is in fact not new in the bibliometric research. As early as 1999, McCallum proposed a mixture author model with the mixture weights for different topics fixed [6]. Then, in 2004, Rozen-Zvi proposed author topic modeling [1], which is the integration of LDA and the author model. It aims at extracting information about authors and topics from large text collections simultaneously. Since then, author topic modeling has been widely used in applications such as bibliometrics analysis [7], information extraction [8], social network analysis [9] named entity recognition [10] and MeSH indexing interpretation [11].

However, modeling author-topic-word relations in TRS has not been attempted. Given the large increase in tobacco-related research, which the FDA has regulatory authority over tobacco, author topic modeling can help the field better understand the nature and scope of research already underway, and serve as a means of fostering interdisciplinary science that is needed to inform tobacco policy [12]. Moreover, our work aims at filling this gap in order to extend author topic models into medical corpus analysis.

## Materials and methods

### Author topic modeling (ATM)

ATM aims at extracting information about authors and topics from a large text collection simultaneously. It is a class of Bayesian graphical model for text document collections represented by bag-of-words. In standard LDA, each document in the collection of *D* documents is modeled as a multinomial distribution over *T* topics, where each topic is a multinomial distribution over *W* words and both sets of multinomial are sampled from a Dirichlet distribution.

Different from LDA, ATM incorporates authors by adding one more variable, which is uniformed assigned by a set of authors, an observed set in some corpus. As in LDA, a topic is chosen from a distribution over topics specific to that author, and the word is generated from the chosen topic.

To learn the model parameters, we use Gibbs sampling where the *equation* for author topic modeling is,

$$ P\left({z}_{id}=t,{y}_{id}=a\Big|{x}_{id}=w,\ {\boldsymbol{z}}^{\neg id},{\boldsymbol{y}}^{\neg id},\mathrm{A},\upalpha, \upbeta \right)\propto \frac{N_{wt,\neg id}^{WT}+\beta }{{\displaystyle {\sum}_{w\hbox{'}}}{N}_{w\hbox{'}t,\neg id}^{WT}+W\beta}\frac{N_{ta,\neg id}^{TA}+\alpha }{{\displaystyle {\sum}_{t\hbox{'}}}{N}_{t^{\hbox{'}}\alpha, \neg id}^{TA}+T\alpha } $$

where, *α* and *β* are Dirichlet priors for topic distributions, *z*_*id*_ = *t* and *y*_*id*_ = *a* are the assignments of the *i*th word in document *d* to topic *t* and author *a* respectively and *x*_*id*_ = *w* indicates that the current observed word is word *w. N*^*TA*^ represents the topic-author count matrix, where ${N}_{ta,\neg id}^{TA}$ is the number of words assigned to topic *t* for author *a* excluding the topic assignment to word *w*_*id*_. Similarly, *N*^*WT*^ is the word-topic count matrix, where ${N}_{wt,\neg id}^{WT}$ is the number of words from *w*^th^ entry in the vocabulary assigned to topic *t* excluding the topic assignment to word *w*_*id*_. Finally, ***z***^¬ *id*^ and ***y***^¬ *id*^ represent the vector of topic assignments and vector of author assignment in all corpus except for the *i*^th^ word of the *d*^th^ document respectively.

Following the same convention, the posterior distribution of *θ*_*ta*_, the topic distribution of each document and *ϕ*_*wt*_, the topic distribution of each word, can be estimated with the following equations where *D* refers to the corpus.

$$ \begin{array}{l}{\theta}_{ta}=p\left(t\Big|a,d\right)=E\left[{\theta}_{ta}\Big|{\boldsymbol{z}}^{\neg id},D,\alpha \right]=\frac{N_{ta,\neg id}^{TA}+\alpha }{{\displaystyle {\sum}_{t\hbox{'}}}{N}_{t^{\hbox{'}}\alpha, \neg id}^{TA}+T\alpha}\\ {}{\phi}_{wt}=p\left(w\Big|t\right)=E\left[{\phi}_{ta}\Big|{\boldsymbol{z}}^{\neg id},D,\beta \right]=\frac{N_{wt,\neg id}^{WT}+\beta }{{\displaystyle {\sum}_{w\hbox{'}}}{N}_{w\hbox{'}t,\neg id}^{WT}+W\beta}\end{array} $$

This model can be understood as a two-stage stochastic process. An author is represented by a probability distribution over topics, and each topic is represented as probability distributions over words.

### Data gathering and preprocessing

In order to obtain a comprehensive collection of all tobacco-related research, we collected publications from two sources. The first source is 300 tobacco-related keywords developed from the FDA CTP’s key research priority and interest areas as outlined on the various TRS Funding Opportunity Annoucements’s (FOA’s), released in partnership with NIH, since the passage of the FSPTCA in 2009. The final search term list was reviewed and refined by FDA CTP and NIH ODP staff, bibliometric and tobacco research experts. The second data source is the publications from the 131 principle investigators of TRS grants funded by the CTP through the NIH’s Tobacco Regulatory Science Research Program (TRSP) (http://prevention.nih.gov/tobacco/portfolio.aspx). Among the TRS PIs are 65 investigators that are part of the Tobacco Centers of Regulatory Science (TCORS), a large 14-center initiative that serves as the flagship for the TRSP. Since each article can have multiple authors, the author set considered in this work includes PIs plus the last author of the paper. The final author set includes 2,740 authors. The document set includes those MEDLINE citations with abstract available, resulting in 167,196 and 8,800 abstracts respectively. We refer to the first dataset, pulled using TRS keywords, as the KWSet and the second dataset, using publications from TRS grantees, theTRSAwardeeSet.

For each document, we removed stop words using a stop word list available at Mallet software package [13]. We then stemmed the words by applying the potter stemmer [14] and words with occurrence lower than 2 are discarded. We further filtered out words based on Term Frequency-Inverse Document Frequency (TF-IDF), where words with high document frequencies and relatively insignificant for single document were removed.

### Evaluation

The evaluation of ATM, as other topic models, can be conducted from two aspects, topic interpretability and topic coherence. Interpretability refers to how much degree human beings can understand topics generated by a topic modeling. It is often regarded as one of important measures to tell how good an unsupervised model is th more on quality. In this paper, we give a detail analysis on what each topic represents and whether they match areas TRS focuses and if not so matching, what rational we can find out. For topic coherence, on the other hand, we employ quantitative measures to make estimations. Both perplexity and pointwise mutual information (PMI) are employed for this purpose. Perplexity measures the degree how fit the topic model is to the training data while PMI measures topic coherence by calculating conformation measures of top *N* words used to represent each topic. The perplexity is defined as the integrating out of all latent variables, namely, $perplexity\left({D}_{test}= \exp \left\{\frac{{\displaystyle {\sum}_{d=1}^M} logp\left({w}_d\right)}{-{\displaystyle {\sum}_{d=1}^M}{N}_d}\right\}\right)$. The lower the score, the better the model fitness. The PMI-based coherence measure is calculated by,

$$ C=\frac{2}{N.\left(N-1\right)}{\displaystyle \sum_{i=1}^{N-1}}{\displaystyle \sum_{j=i+1}^N}PMI\left({w}_i,{w}_j\right) $$ and $$ PMI\left({w}_i,{w}_j\right)= log\frac{P\left({w}_i,{w}_j\right)+\epsilon }{P\left({w}_i\right)P\left({w}_j\right)} $$

where *P*(*w*_*i*_, *w*_*j*_) is the probability of *w*_*i*_ and *w*_*j*_ co-occur in the whole corpus and *ϵ* is added to avoid logarithm of zero.

### Temporal trend analysis on key words

Temporal analyses of research topics can reveal interesting trends and provide guide for future endeavors. According to the FDA, there are four key areas in TRS. They are *cigars*, *smokeless tobacco*, *e-cigarettes* and *tobacco product characteristics*. Therefore, in order to achieve this goal, we extracted publications, which were published from 2000 to 2013, corresponding to the four key areas from the larger KWSet. Then we divided all abstracts by year and ran author topic modeling on them respectively. Next, we calculated the proportion of key words in all the topics with ${\displaystyle \sum_{k=1}^K}p(k)*p\left(w\Big|k\right)$ where *K* is the total number of topics (*K* = 400 as determined in last section), *w* is the key word, *p(k)* is the proportion of each topic and *p(w|k)* is the probability of the key word in *Topic k*.

## Results

### Articles’ yearly distributions

**Figure 1:**
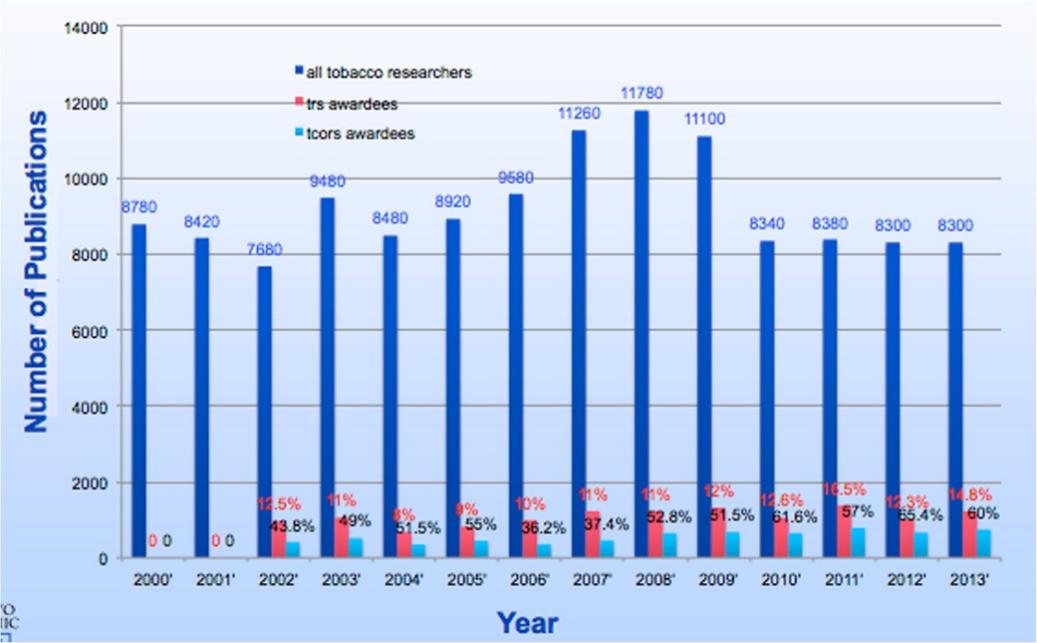
**Diversity of TRS publications against annual counts X-axis is the year and Y-axis is the number of publications.**

**Figure 2:**
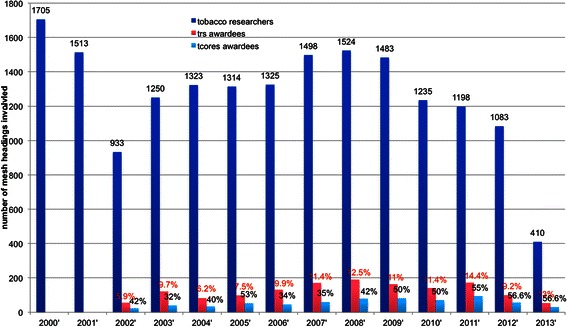
**Mesh diversity of TRS publications annually X-axis is the year and Y-axis is the number of mesh headings involved.**

Figures 1 and 2 show the yearly distributions of all tobacco related publications. Figure 1 shows that from the year 2000 onward, there were about 10,000 newly published articles related to tobacco regulatory science each year. Among them, TRS awardees contributed about 10%. TCORS awardee publications made up half of TRS awardee contributions.. The total number of articles have seen a slight increase from year-to-year over the past 10 years, with the exception of 2007 to 2009, where there was a large jump in numbers. Yet, after 2009, the number becomes stable. This may be related to the short-term grants funded during economic stimulus efforts (ARRA grants) [16], which had some different publication and research dissemination stipulations than more traditional grants.

We also investigated the distribution based on Mesh headings [17], which is a comprehensive controlled vocabulary for the purpose of indexing journal articles and books in the life sciences (illustrated in Figure 2). The general trend looks quite similar to that in Figure 1, in that there were slight increases from year-to-year. However, a few differences can be observed as well. Among the literature retrieved using the keyword search queries, based on CTP research interest areas, a more diverse range of research topics can be observed over time. Due to this large array of diversity, only 10-14% are from TRS-funded researchers, with TCORS awardee publications again constituting half of TRS researcher contributions.

### Articles’ journal distributions

As the first step of bibliometric analysis, we made a simple count of which journals TRS researchers usually publish their articles. This step can be regarded as a compensations for author-topic modeling because we assume that the journals can associate the contexts of the articles to a large degree.

### Top TRS journals from PUBMED keyword data set

The PubMed data set includes 167,196 publications from 7,134 journals. However, there are 1,824 journals from which there was only one article in the TRS keyword data set. In addition, we found that there are 5,146 journals from which there were fewer than ten articles. This indicates that it is likely these journals do not traditionally cover topics related to TRS research. Interestingly, the number of journals from which more than 100 articles were selected is 306, a much smaller and more manageable pool of potential publication outlets for TRS research. Together, those 306 journals published 155,512 of the articles in the TRS keyword data set, or 93% of all publications in the data set; so almost all TRS publications in the past have occurred in these 300 or so journals. Inspecting the top 30 journals publishing TRS articles (see Table 1 where we listed numbers of publications and their ratios for KWSets, TRSAwardeeSet and TCORAwardeeSet), we find that most of the journals topic areas are related to toxicity, biochemistry, nicotine, environments, pharmacology and health.

**Table 1:** **Top journals for KWSet, TRS**

1	Journal name	KWSet	KWSet ratio	TRS	TRS ratio	Tcores	Tcores ratio
2	Nicotine & tobacco research	2142	0.07	540	0.24	241	0.26
3	The journal of biological chemistry	1953	0.06	71	0.03	24	0.03
4	Biochemistry	1407	0.05	54	0.02	18	0.02
5	Journal of the American Chemical Society	1353	0.04	53	0.02	0	0
6	Mutuation research	1204	0.04	38	0.02	16	0.02
7	Bulletin of enviornment contamination and toxicology	1159	0.04	0	0	0	0
8.	Toxicology and applied pharmacology	1118	0.04	38	0.02	0	0
9.	Enviornment science & technology	1095	0.04	64	0.03	20	0.02
10.	The Science of the total enviornmnet	1084	0.04	36	0.02	17	0.02
11	Biochimica et biophysica acta	1082	0.04	0	0	0	0
12	Carcinogenesis	1063	0.03	119	0.05	21	0.02
13	Journal of hazardous materials	1024	0.03	0	0	15	0.02
14	Chemosphere	982	982	0.03	0	0	0
15	Psychopharmacology	953	0.03	124	0.05	64	0.07
16	Inorganic chemistry	948	0.03	47	0.02	16	0.02
17	The Journal of pharmacology and experimental therapeutics	932	0.03	78	0.03	34	0.04
18	Talanta	914	0.03	0	0	0	0
19	Cancer research	896	0.03	91	0.04	17	0.02
20	Biochemical and biophysical research communications	880	0.03	0	0	14	0.02
21	Proceedings of the National Academy of Sciences the United States of America	872	0.03	56	0.02	24	0.03
22	Toxicology letters	843	0.03	0	0	0	0
23	Enviornmental health perspectives	838	0.03	49	0.02	18	0.02
24	Biochemical pharmacology	814	0.03	0	00	0	0
25	Toxicology	807	0.03	0	0	0	0
26	Pharmacology, biochemistry, and behavior	762	0.02	58	0.03	28	0.03
27	European journal of pharmacology	748	0.02	0	0	0	0
28	Brain research	743	0.02	34	0.01	19	0.02
29	Enviornmental pullution (Barking, Essex: 1987)	743	0.02	0	0	0	0
30	Applied and enviornmental microbiology	740	0.02	39	0.02	27	0.03
31	Journal of bacteriology	736	0.02	33	0.01	0	0
32	Addiction (Abingdon, England)	0	0	71	0.03	32	0.03
33	Addictive behaviors	0	0	91	0.04	41	0.04
34	American journal of public health	0	0	0	0	16	0.02
35	Cancer epidemiology, biomakers & prevention	0	0	91	0.04	29	0.03
36	Chemical research in toxicology	0	0	67	0.03	0	0
37	Drug and acohol dependence	0	0	85	0.04	43	0.05
38	Ecperimental Experimental and clinical psychopharmacology	0	0	0	0	19	0.02
39	Journal of neurochemistry	0	0	0	0	15	0.02
40	MMWR, Morbidity and mortality weekly report	0	0	64	0.03	0	0
41	Neuropharmacology	0	0	38	0.02	18	0.02
42	Neuropsychopharmacology	0	0	40	0.02	22	0.02
43	Science (New York, N.Y.)	0	0	33	0.01	15	0.02
44	The Journal of neuroscience: the official journal of the Society for Neuroscience	0	0	0	0	19	0.02
45	Tobacco control	0	0	79	0.03	25	0.03
46	total	30835	1	2281	1	927	1

### Top journals covered by TRS investigators

Top journals covered by all TRS investigators are almost identical to those of the PUBMED keyword data set (see Table 1). But differences are also evident. Journal coverage of TRS investigators is more focused on those related specifically to tobacco. For example, *Tobacco Control* is one of the main journals (top 8) in the top journal list of TRS investigators, though the top journal among the two lists is the same. In addition, *Addictive Behaviors* is also in the top 10. From this, we can see from a different perspective what CTP funded researchers concentrate on.

### Top journals covered by TCORS investigators

The top journals covered by TCORS investigator publications are quite different from the journal coverage of the larger TRS investigator group, with a few subtle differences in the ordering and prominence of a few journals. For example, Brain Research is much more prominent (top 16) among the TCORS journal list, while it ranks 27^th^ among the larger TRS group.

### Author topic modeling experiment and topic coherence evaluation

We ran the author topic modeling developed by Steyvers and et al. [15] on both the KWset and the TRSAwardeeSet. The topic number, *T* is determined by the grid search and comparison of perplexity defined in last section. Similar to LDA, estimation of topic distributions of words was evaluated with Log-likelihood score of the posterior distribution of words given topics, one of the standard criteria for generative model evaluation. It was found that the best perplexity for KWset was the lowest when T is 400 and the best for TRSAwardeeSet was 20 respectively. The hyperparameters *α* and *β* were fixed as 50/T and 0.01 respectively and according to the data size, the iterations for both data were 1000 and 50 respectively. The PMI evaluation for the coherence of the resulting topics yields 65% and 70% on average. These results were basically consistent with those reported for domains in news, social media or computer science. This shows that ATM can be adapted in medical fields. In order to confirm this quantitaive evaluation, more quality analysis is done in the following sections.

### Topic interpretations

**Figure 3:**
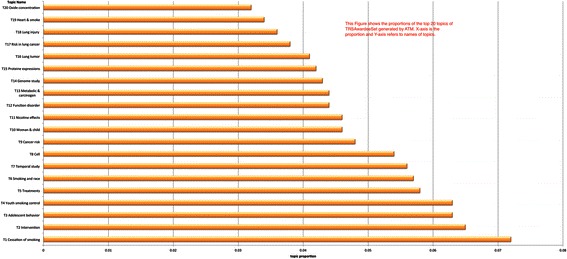
**Topic Proportions for the 20 topics of TRSAwardeeSet The X-axis is the topic number and the annotated topic name and the Y-axis.**

**Figure 4:**
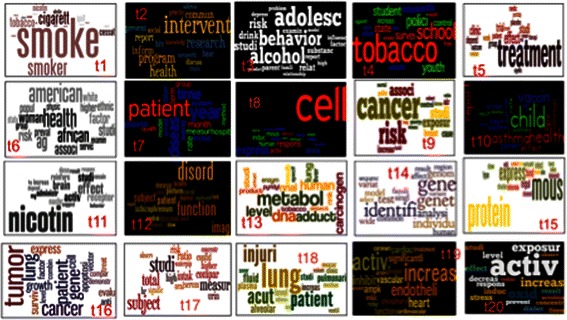
**Word cloud for 20 topics of TRSAwardeeSet For TRSAwardeeSet**, **we use wordle to generate word cloud for top 20 words of each topic and then put all word clouds into one slide for visualization.** The 20 topics are order from left to right and from up to down. The size of words reflect the proportion of each word in that topic. Note, the word is not really word, instead, a stem.

Figure 3 shows the ordered proportion of the 20 topics for the TRSAwardeeSet and Figure 4 shows the word clouds of the top 20 words for each topic. In order to find out what each topic is focused on, we assign each topic a name based on the top 20 words. The naming in this paper is done manually by domain experts and we are implementing a semi-auto labeling algorithm. The auto-labeling methodology and results will be reported in future work.

We can see that the 20 topics have comparatively balanced distributions ranging from 0.034 to 0.071. One thing worth noting is that some of the topics may be somewhat irrelevant to TRS. For those relevant to tobacco research, the topics derived have a broad diversity as discussed in the following. Top words included in the most prominent topic (namely, T1) from the dataset are *Smoke, cigarette, cessation, abstinence, control, measure*. T2, which is the second most prominent topic, contains words like *intervention, health, program, network, base, train, social, prevent, address, support and community*. T3 focuses on adolescent-related topics, including *alcohol*, *family relationship* and *behavior*. T4 is similar to T3, but emphasizing more on social elements, including *school*, *law*, *industry* and so on. Those topics suggest policy-making and social studies such as preventing teenager-smoking related research are one of the major trends in TRS research. *Treatment* is most dominant word in T5. Among them, most of words are quite relevant to this keyword. These words suggest that research on clinical practice of smoking related diseases is also tackled by TRS researchers. T6 evidently clusters research on ethnic, gender, age and surveys of smoking revealed by words *American, African, white, group, population, ethnic, woman* and *age*. In contrast, T7 talks about temporal study of smoking-related diseases since temporal words like *time, year, month* and clinical words like *assess, measure, average, quantity, disease* and *datum* are seen there with a good proportion.

Topics from T8 to T20 are all related to direct clinical studies because from now on, we can see there are quite a few domain-specific terms among each topic and understandably, they occupy fewer proportions due to the domain constraints. But the fewer portions do not mean that they are less important for the modeling. On the contrary, they can show how discriminative the author topic modeling can be. For example, the word *cell* is the dominant word in T8. Surrounding it are *words mouse, receptor, express, airway pressure, vitro, response, inhibition, epithelial* and *mediation*. Therefore, this topic talks more about experiments on the influence of smoking on cells. Their proportions are relatively smaller. T9 is obviously discussing relationships between smoking and cancers where *cancer, risk, association, control cohort, air, lung* and *genotype* are the prime terms. Furthermore, *pollution, exposure, woman* and *breast* also suggest that the indirect influence of smoking is included in this topic.

As seen, the core of T10 is *child* with smoking related terms *asthma*, *screen*, *vaccine* and *HPV*. As we mentioned above, all of those topics are more specialized. Without domain knowledge, it can be hard to understand why *HPV* is related to smoking. In fact, according to Troy et al. [18], a case–control study of childhood passive smoke exposure (CPSE) is with human papillomavirus (HPV) infection. *Nicotine* in T11 has the highest proportion, as much as 5%. It is not hard to imagine that this topic should mainly discuss nicotine and its effects. Words such as *cocaine, brain, response, behavior, kg, mg, reinforce* and *nach* prove this. T12 seems to mainly study the disorder brought by smoking and their correlations. It is composed of words including *disorder, function, schizophrenium, depression, correlation, discrimination* and so on. T13 comprises of a couple of rarely seen terms, such as abbreviations, *DNA*, *NNAL* (urinary total 4-(methylnitrosamino)-1-(3-pyridyl)-1-butanonol, which level can be affected by smoking) and *nnk* (4-(Methylnitrosamino)-1-(3-pyridyl)-1-butanone, one of the most prevalent and procarcinogenic compounds in tobacco), organic chemical elements, *pyridyl* and enzyme cancer terms, *carcinogen*, *adduction*, body and function terms, *lung*, *liver*, *urinary*, *metabolic* and so on. Among them, metabolic is the leading term unifying all of them. The majority of these topics are related to the harmful and potentially harmful constituents of tobacco products listed as one of the ten interest areas of TRS that have been highlighted by the FDA. *Gene, genetic, genome sequence, individual, variant* and *identify* in T14 show that this research topic on tobacco is from the genetic perspective while *protein, mouse, regulation, binding, express* and so on in T15 more from regulation and binding mechanism of the protein.

T16 is also about cancer. However, different from T9, it focuses on lung cancer and treatment. The corresponding cell apoptosis can be indicated from words like *survival, anti, treat, apoptosis* and so on. T17 seems to mainly be related to the medical absorption since we can see words like *intake, concentration, ratio, oral, serum, urinary, waterpipe* and *others* to name a few. T18 also talks about lung, but it is not about lung cancer. Instead, it is more about the general aspects of lung injury since ventilation, plasma, injury, acute and edema are there. Although smoking affects lung so much, T19 tells us that heart diseases are quite related as well where *heart, cardiovascular, cardiac, vascular, endothelial, phosphoric, artery,* and *coronary* are high frequent terms. According to Wheat et al. [19], inhalation of tobacco increases apoptosis and suppresses the VEGF-induced phosphorylation of Akt and endothelial nitric oxide synthases in the aorta. The last one, T20, looks like associating smoke and diabetes through similar mechanism in T19. Acrolein, an element rich in tobacco, is the main element, which prevents the nitric oxide, to lead to smoking-caused diseases. All those 20 topics, we can say that most of them have a good alignment with TRS. This shows that author topic modeling is capable of modeling the topic distributions of the collection.

### Author-topic relations

**Figure 5:**
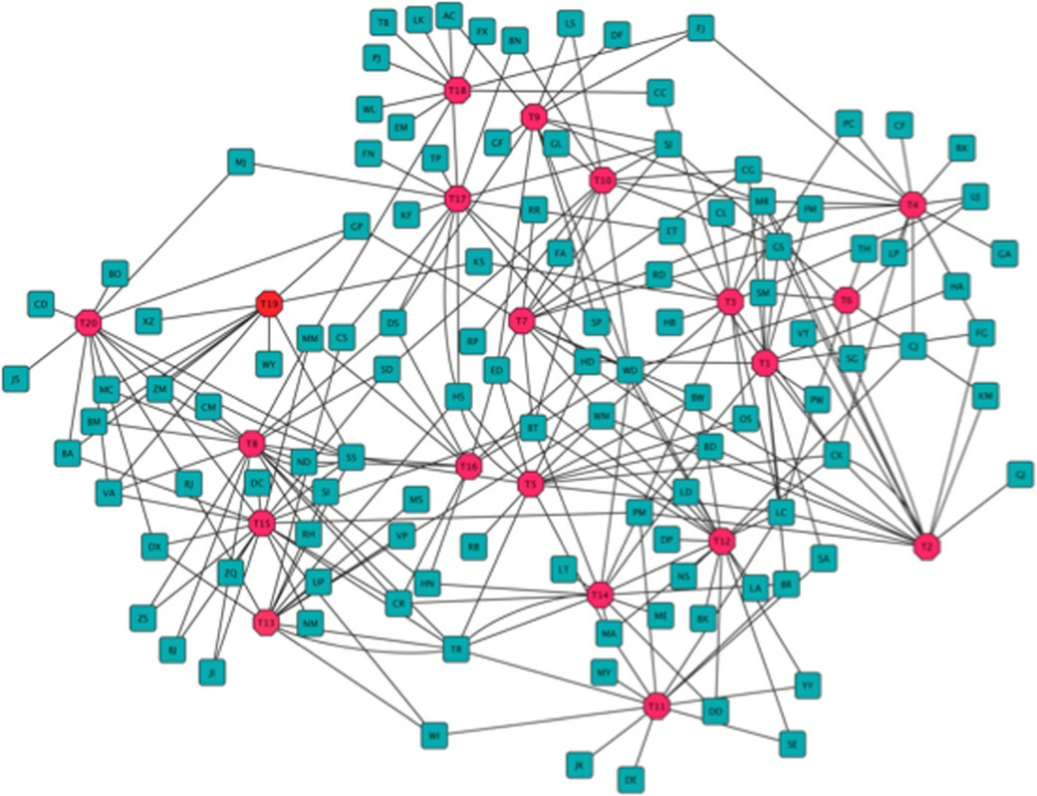
**Author topic network for the 20 topics of TRSAwardeeSet For 20 topics, we build a network against its top 20 authors so that we can see clearly the productivity and diversity of authors and the closeness between authors (if two author nodes are linked to the same topic node, we may say that they have common interests).**

Figure 5 is a network with each topic as the hub (the red octagons) and authors form nearest neighbors of each topic if their research involves that topic (the green plate). Figure 5 demonstrates the results of the top authors (if the author had more than 0.01 portion of articles in that topic, they were counted as a top author. The portion is selected based on the observation that 0.01 of more than 8800 articles, namely, 88 articles for one PI, can be regarded to be quite productive in research) and their associated topics. For better visualization, initials for authors are used to represent them (see Additional file 1 for the corresponding full names. The correspondence of initials and full names is seen in the supplement. Based on this network, it is found that the top 5 authors in each topic are the prime principle investigators in corresponding topics. For example, Hatsukami D, Cummings K and Eissenberg T, the top three ranked in T1, are all senior tobacco researchers who mainly focus on tobacco addiction characterization, reduction and/or treatment. Meanwhile, as shown in the network, there are connections between topics. That means that many authors’ research areas cover more than one topic.

**Figure 6:**
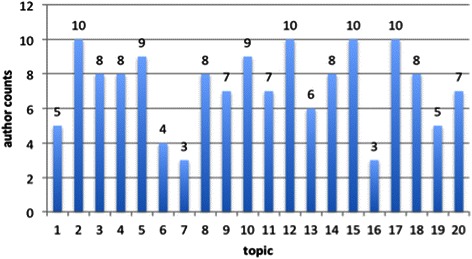
**Author counts in topic maximum.** The X-axis is the topic while the Y-axis is the number of authors who work on some topic.

At the first glance, Figure 6 is similar to Figure 5, aiming at showing how many authors appear in one topic. But the main goal of Figure 5 lies in illustrating who are top authors in a topic while Figure 6 illustrating some topic is the most studied one for some author. For example, the 3 counts for T7 in Figure 6 indicates that there are three authors whose highest portion are in T7 while the 14 nearest neighbors around T7 in Figure 5 show that 14 authors have portions larger than 0.01 in their research for topic 7. Figure 6 shows that T2, T12, T15 and T17 are the most studied topics since for each of them, 10 author published large number of articles on them. This trend does not align with that of topic proportion. To a large degree, we can say that the topic proportion shows that how many researchers are studying what topics while authors’ counts reflected in Figure 6 show that which topic has been intensely studied by a few researchers. If we count T5 and T9 (there are 9 authors respectively), the data suggests that tobacco prevention and treatment are popular topics among those researchers.

Another interesting thing is to look at co-occurrence of authors among multiple topics (for simplicity, we only consider two). It can reflect two aspects, one on the closeness of two topics (the two or more can be subtopics of a big topic) and the other on interactions of two topics (they may not be related but depend on each other).

It is found that T15 and T8 co-occur together 10 times, ranking the highest. This indicates that 10 authors study both topics. Both topics involve *genetic expressions*, *cell*, and *protein*. The combination of T16 and T8 follows closely where topic 16 is about *lung tumor* study from *gene* and *cell* level. The topic dependence relation can be illustrated by the large number of topics co-occurring with T2 (*intervention*). This topic is not really funded by the FDA’s CTP, so why do they have such a high proportion of research (0.065)? If we look at other topics which investigators focus on in addition to T2, we can discover clues. Three topics occurring quite commonly with T2 are T1, T3 and T4 (4 times respectively). These four topics are about smoking cessation, vulnerable populations, and youth initiation and access, all of which are TRS priority areas. The link between *smoking cessation* and *intervention* is interesting, as interventions focusing on cessation are specifically mentioned as not a fundable TRS area. Investigators with this topic pair, which is common in tobacco control research in general, may be looking at other related topics that do fall under the TRS scope, such as *nicotine reduction*, *consumer perception* (of certain products as a cessation aid) and *effective communication strategies*. In addition, T7 (*temporal study*) co-occurs with T2 three times as well. This connection between *temporal study* and *intervention* would be a necessary one, as intervention research requires studies across time.

### Topic clusters based on authors

**Figure 7:**
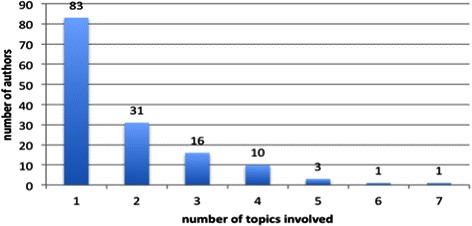
**AT involvements.** The X-axis is the number of topics involved while the Y-axis is the number of authors who are working on how many topics.

If we look at authors and topics they are assigned, we see cases for two extremes in terms of involvement with a diversity of topics. Figure 7 shows that 83 top authors, in fact, focus on only one topic. A small number of authors have high topic involvement, meaning involvement in many topics. The highest one is *Williams D* who studies 7 topics. The next four are *Srivastava S* (6 topics), *Glantz S* (5 topics), *Baker T* (5 topics) and *Elashoff D* (5 topics) respectively. *Williams D*, as the most diverse researcher, is in fact a leading social and behavior scientist focusing on public health [20]. His research has enhanced the understanding of the complex ways in which race, racial discrimination, socioeconomic status and religious involvement can affect physical and mental health. His topics in the tobacco regulations cross from intervention study, health and race, gender, age, functional disorder and genetic analysis and so on. *Glantz S* is American Legacy Foundation Distinguished Professor of Tobacco Control at the University of California – San Francisco whose research focuses on the health effects of tobacco smoking and who is active in the nonsmokers’ rights movement and has advocated for public health polices to reduce smoking. His research topics include T1, T2, T4, T7 and T10, which quite match his research focus.

*Baker T* is involved in T7, T10, T12, T14 and T15 while *Elashoff D* in topic T5, T7, T9, T12 and T16. They have two overlapping both with T7 and T12 assigned to them. Both of them seem to study topics related to treatment of smoking related diseases. What *Elashoff D* is studying is more cancer related. The topics both of them share are more general aspects like temporal study, function disorder and genetic tests. The remaining topics *Baker T* has, like T10, T14 and T15 involve smoking cessation, intervention, influences on children and protein binding and regulations. On the other hand, *Elashoff D*’s remaining topics including T5, T9 and T16 are all either cancer-related or organ-injury relevant. In *Baker T*’s webpage [21], it states that *Baker T* concentrates on tobacco-dependence treatment and outcomes. He and his team are not only looking at smoking cessation, but also determine how quitting affects the person’s physical health, mental health, quality of life and social interactions. Then, *Elashoff D*’s research include statistical analysis of high-throughput microarray, biomarker discovery and validation studies. Meanwhile, he has extensive working on cancer related projects with collaborations in oral, lung, prostate, breast and skin cancers [22]. It seems that those descriptions confirm what we have found from those topics.

As mentioned before, topics discovered are not necessarily all primarily about tobacco and nicotine in this work. Instead, it focuses on finding the interactions between authors, topics and words and what trends can be traced under the frame of TRS. Observing along this thought, we found connections between tobacco and other related topics unique to TRS research. For instance, *Srivastava S*, a project lead on a TRS center grant, is not primarily a tobacco researcher. Instead, he is faculty in an environmental cardiology department. His topic profile includes T6, T8, T13, T15 and T16. From his webpage, we found that his research priority is toxicity, which can explain the connections of the 6 topics: all of them are less or more related to his priority. It is also a topic area prominently featured in the FDA’s TRS priority and interest areas.

On other extreme, there are a few PIs who are only assigned one topic. One of them is *Delnevo C* whose topic is T6, which is about ethnic, gender and age related study of smoking. In the website, it says that his research interests are clinical prevention services, tobacco control and survey research methods. Another one is *Donny E* whose topic is T11, which is about the nicotine effects. In his webpage, it says that nicotine reinforcement, regulation of tobacco and implications for healths are his primary research interests. Likewise, *Farrelly M* is a leading expert in tobacco control and policy interventions, for youth in particular. The only topic assigned to him is T4, exactly matching his interests.

### Top topic clusters

**Figure 8:**
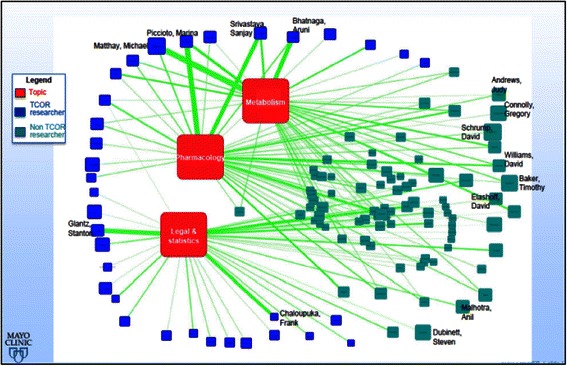
**A sample of author topic relation network (3 topics).** This figure aims at highlighting three top topics where the proportions of TCOR PIs and non-TCOR PIs show clear contrast.

Figure 8 aims at highlighting three top topics where the proportions of TCOR PIs and non-TCOR PIs show clear contrast. The top topic clusters for all TRS investigators are *metabolism*, *pharmacology*, and *legal & statistics*, where the three top topic clusters are depicted in red. In the author topic network, topics are connected to authors who publications are linked to those terms. The size of the author nodes and the edges connecting them to the three topics reflect the importance of the authors and the contributions to the three topics respectively. For example, *Matthay, Michael* is a prominent author in the network since the node representing him has the largest size. His main research is on *metabolism* since the edge to it is the thickest. He is not linked to either *pharmacology* or *legal & statistics,* though, as *Piccioto, Marina* is (i.e. she is linked to all three topics); although the latter is comparatively less prominent than the former. Both of the above authors are TCORS investigators (the blue nodes).

**Figure 9:**
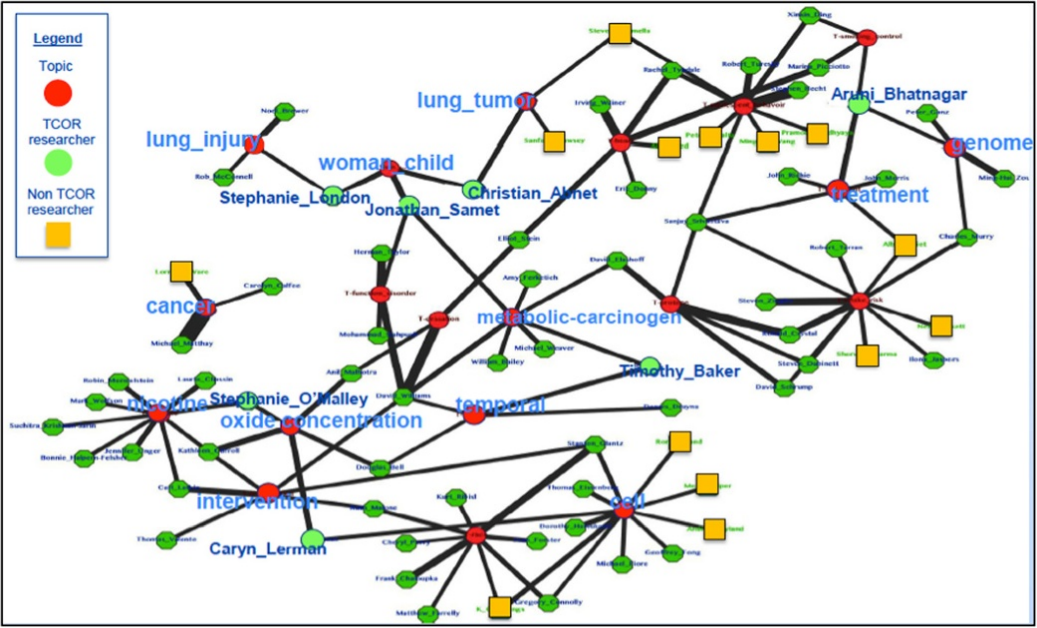
**Author topic modeling for TRS PIs.** The network of topics against TCOR PIs and non-TCOR PIs. It aims at showing what research interests for TCOR PIs and non-TCOR PIs and also how much overlapping the group of researchers. It is a compenstaion for Figure 8.

Also depicted in this figure are other TRS researchers (the green nodes). One key take-home point from Figure 8 (Figure 9 can be a compenstation for Figure 8) is that, while there are common research interests among the TCORS investigators, there is also a large body of expertise among the other TRS grantees as well and that both groups of investigators should be finding ways to link with others around common or shared TRS research interest.

### Temporal trend of key words

**Figure 10:**
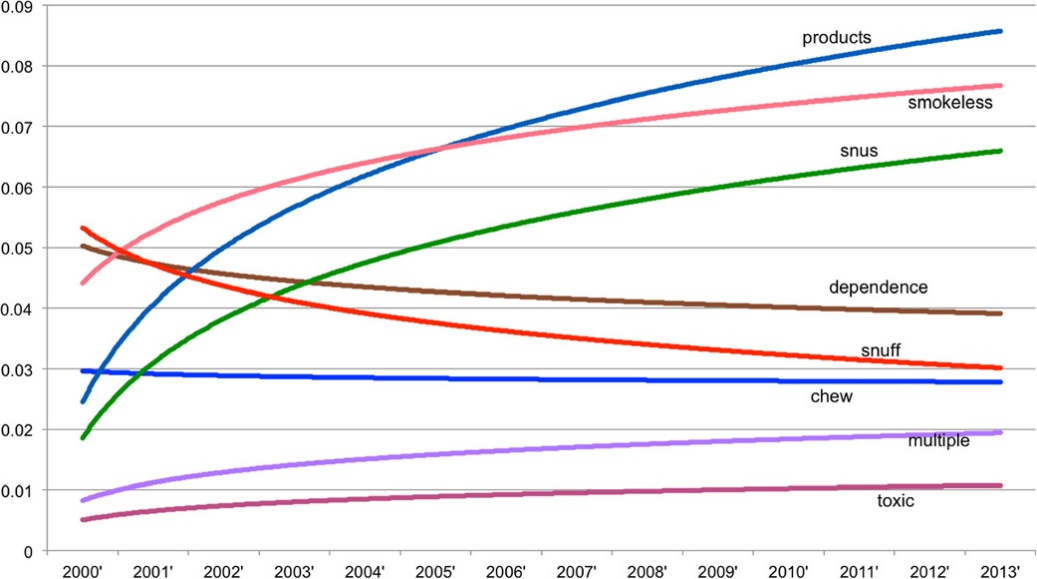
**Smokeless tobacco temporal trend.** The X-axis is the year while the Y-axis is yearly proportion of key words of smokeless tobacco.

**Figure 11:**
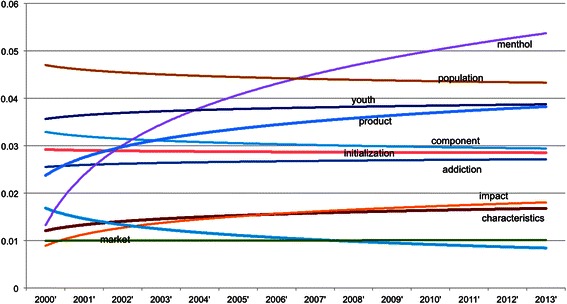
**TPC temporal trend.** The X-axis is the year while the Y-axis is yearly proportion of key words of TPC.

**Figure 12:**
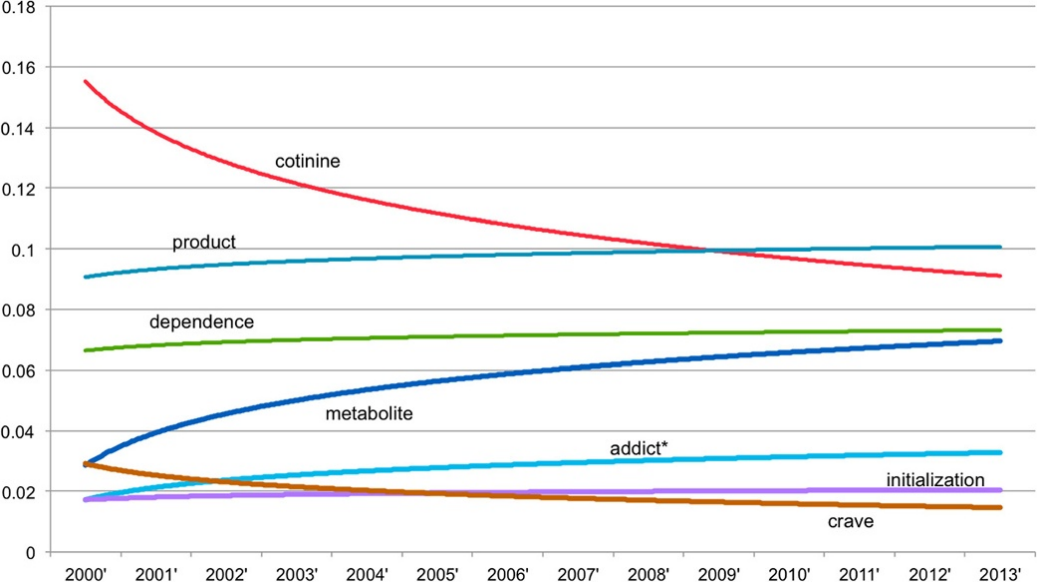
**Cigar products temporal trend.** The X-axis is the year while the Y-axis is yearly proportion of key words of cigar products.

**Figure 13:**
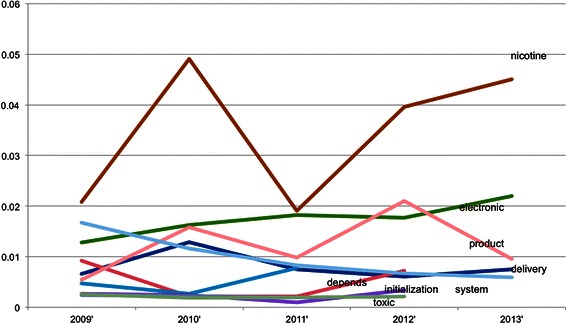
**E**-**cigarettes temporal trend.** The X-axis is the year while the Y-axis is yearly proportion of key words of E-cigarettes.

Above analyses are based on the pool of MedLINE abstracts of TRS PIs without distinguishing publication time and areas. For If we The trend can be seen in Figure 10, Figure 11, Figure 12 and Figure 13, where the x-axis is the list of years while the y-axis is the proportion of key words in the data.

For the study of smokeless tobacco in Figure 10, the trends are not overwhelmingly informative. However, two key terms, *snus* and *smokeless*, are both steadily increasing over the time period in similar trends. While another term, *snuff*, decreases relatively sharply over the same time period. *Chew* shows a gradual decreasing trend. This shows an increased research interest over the past 10 years in the alternative smokeless tobacco product, *snus.*

Figure 11 shows the trends for the top terms related to tobacco product characteristics. The trend for the term *menthol* is the most prominent one for this figure. *Menthol* shows a clear increasing ratio among the tobacco products characteristics terms, where the overall trend for all terms appears to be a decreasing one. The popularity of *menthol* demonstrates the increasing focus on flavorings as a research area for tobacco product characteristics [23,24] in recent years.

For cigar products, the top seven key terms are displayed in Figure 12. Interestingly, cotinine, the most prominent term, decreases continuously from around 16% to 9% over the 13 years. Meanwhile, the less prominent term metabolite, increases steadily over the same time period from 3% to 7%. This seems somewhat counterintuitive, as cotinine is a metabolite of nicotine. This could simply indicate a change in the preference of terms from the specific to the more general. It could also indicate the decline in the use or study of cotinine as a measure of nicotine use. Other types of cigar products, such as little cigars and cigarillos, are not yet prominent enough in the literature to be in the top cigar-related terms.

Electronic cigarette terms are shown in Figure 13 and likely because of the recent emergence of these products, this figure doesn’t show any consistent or clear trends. One key point for this topic, though, is a bit different from the others. This analysis highlights the need for some consistency and consensus on what to call new and emerging tobacco products, like electronic cigarettes, in the literature. There are several different terms used and because of this diversity in terminology referring to basically the same product, there are larger implications for the research. For example, if different investigators are using different key terms or measures for the same products, it becomes hard to look across a given topic or field, develop standards, and conduct consistent reviews and meta-analyses of the literature.

## Discussion and conclusions

### Summary

In this work, we employed author topic modeling to conduct a bibliometric analysis on the publications of principle investigators on tobacco. We only reported topic interpretations and observations for the TRSAwardeeSet, as our primary interests were the TRS investigators. In fact, the KWset were diverse in both author and topics, and thus a more in-depth exploration is needed to understand this dataset. Nonetheless, we did temporal trend analysis based on the results of ATM for KWset. It showed us how the significance of key words in different topics evolved over time.

Author topic modeling has been shown to be an effective approach in modeling corpus of computer sciences as well as more general ones, like publicly available emails, collections of diverse research articles. No research is done in modeling a constraint domain like tobacco regulations yet. The results show that this approach can efficiently cluster collections of articles into discriminative categories without any supervision. More interestingly, it can associate topics to authors in a high accuracy. This indicates that we may incorporate author topic modeling into author identification systems to infer the identity of an author of articles using topics generated by the model.

The relevance of this analysis to TRS is multiple folds. First, this analysis is a ‘proof of concept’ that it can be beneficial to assess the change over time in TRS as new projects are funded and collaborative science in this area changes. This is particularly important because the FDA must use the data from funded research to inform their regulatory decision-making, so if there are ‘holes’ in the types of research being conducted or published, a bibliometric analysis with ATM could be helpful for the FDA to make decisions. The results can be used to assess the extent to which new research reflects the funding priorities of the FDA.

Second, ATM outcomes can be used by investigators to assess who is conducting research in a particular research domain in order to foster collaborative science [25,26]. Again, this is very important for the FDA to know given their need to make regulatory decisions. For example, if the FDA is contemplating a regulation that would lead to reductions in nicotine within cigarettes, assessing who is conducting research that can inform that regulatory process is important. Similarly, if the FDA needs to conduct rapid research to address an emerging issue, they can use this type of data to identify likely research teams to carry out that research. Since many issues in tobacco regulatory science require trans-disciplinary science, which cannot be addressed through the research of a single discipline, the ability to assess who is doing relevant research can lead to the development of unique teams that have the best potential to address those complex problems rapidly.

Third, these analyses begin to demonstrate the evolving research productivity of investigators, which we anticipate will occur to a greater extent as publications increase due to FDA funding. For example, we found that ‘cessation’ and ‘treatment’ clustered even though that topic is not really included in tobacco regulatory science. This clustering seems to reflect that some leading scientists who conducted research on tobacco treatment have successfully either shifted or expanded their research focus on tobacco regulatory science. Future analyses can further delineate how scientists transition into tobacco regulatory science research, particularly as a result of new funding, to better understand both the scientific expertise relevant to the TRS field, and also to understand the impact in other fields of scientists following the increased funding in TRS.

By fostering collaborative science in TRS, it becomes possible to speed advances in that science by fostering communication between scientists that can avoiding un-needed duplication and impact decision-making on new science that can benefit regulatory decision-making.

### Limitations and future work

One limitation for this approach is that author topic modeling assumes that the topic distribution of each word in one document is only associated with one of the known authors. As a result, correlations of authors cannot be reflected from words of the same document and instead, must be found across multiple documents, which have the same authors. For large amount of corpus, this may not be a big problem. Nonetheless, this limitation can be overcome if we introduce the topic-author as multiple to multiple. Namely, instead of sampling one author each time, we allow sampling more than one. Then, the topic distribution will be generated by the joint distribution of more than one author. This way, each word will be associated with more than one author and thus, a multiple to multiple word-author interactions will be constructed. This will lead to more complicated inference algorithms. More high efficient optimization algorithms are thus needed in our future work.

The other limitation of our work is the one to one author-word correspondence. Hence, in our future study, we will extend author topic modeling into group author topic modeling. In addition, considering that research topics may change every few years even for the same investigators, it would therefore be reasonable to model temporal changes. One more extension can be that we may build a predictive model based on author topic modeling so that we can assign authors to unknown articles or we can predict what main topics an unknown article is about. Yet another limitation is the lag between publication date and current research activity. Given the rapidly changing nature of research and funding in the area of tobacco regulatory science, it is very possible that investigators have moved into different research domains relative to their publication record. This is particularly relevant in the tobacco regulatory science area because it is a relatively new research domain that has caused some scientists to shift their research focus in order to obtain funding that is specifically relevant to the needs of the FDA. Thus, data from the author topic modeling could provide a misleading perspective on current research activities of scientists.

Besides addressing those limitations, we plan to experiment author topic modeling with domain-specific ontologies or information models instead of only the bag of words. One such ontology is the MeSH indexing widely used in PubMed MedLINE. For articles indexed by PubMed, usually about 10 MeSH terms will be assigned to them so that the reader can easily find the theme. Therefore, those MeSH terms can be utilized as key words in doing author topic modeling or alternatively, MeSH terms can be employed as the author variable so that we can construct a mapping from MeSH term to texts. Beyond, since we have collected a lot of data and explored concepts and relations with the help of ATM, it is possible that we may build our own domain ontology independently from MeSH Indexing for tobacco related research and then align or merge with MeSH indexing for future research on data mining.

Another possible extension is that we will attempt to access full texts rather than only abstracts and meanwhile, construct citation links from the reference section. Enriched by full text and citaiton links, we believe that the correlations of research topics in tobacco regular science can be more fully revealed.

## Additional file

Additional file 1: **Correspondence Author Full Name and Short Name.** The following file is supplement to Figure 5 where we use short names for authors so that better visualzation can be kept. Then, from this table, we can find the full name for each short name.
