# Supplementary material for: A bibliometric analysis on tobacco regulation investigators
Source: BioData Min. 2015 Mar 21;8:11. doi: 10.1186/s13040-015-0043-7 (PMC4432889; doi:10.1186/s13040-015-0043-7)
Supplement: Additional file 1: — Correspondence Author Full Name and Short Name. The following file is supplement to Figure 5 where we use short names for authors so that better visualzation can be kept. Then, from this table, we can find the full name for each short name. [file 13040_2015_43_MOESM1_ESM.doc]

**Additional files**

**Additional file 1 – Correspondence Author Full Name and Short Name.** The following file is supplement to figure 5 where we use short names for authors so that better visualzation can be kept. Then, from this table, we can find the full name for each short name.

| short | full name | short | fullname | short | fullname | short | fullname |
| --- | --- | --- | --- | --- | --- | --- | --- |
| GA | Goldstein, Adam | DE | Donny, Eric | BM | Boehm, Manfred | TR | Turesky, Robert |
| SA | Sved, Alan | KF | Kamangar, Farin | PM | Picciotto, Marina | MR | Mermelstein, Robin |
| VA | Vliet, Albert | DF | Dominici, Francesca | EM | Eisner, Mark | BR | Borland, Ron |
| MA | Mukhin, Alexey | CF | Chaloupka, Frank | WM | Wolfson, Mark | CR | Crystal, Ronald |
| FA | Ferketich, Amy | GF | Gilliland, Frank | PM | Pentz, Mary | RR | Robertson, Rose |
| HA | Hyland, Andrew | FG | Fong, Geoffrey | WM | Wewers, Mary | MR | Malone, Ruth |
| LA | Lees, Andrew | SG | Singh, Gopal | FM | Farrelly, Matthew | DS | Dawsey, Sanford |
| SA | Strasser, Andrew | CG | Connolly, Gregory | KM | Kreuter, Matthew | SS | Srivastava, Sanjay |
| MA | Malhotra, Anil | RH | Reinecke, Hans | NM | Nibert, Max | HS | Heil, Sarah |
| BA | Bhatnagar, Aruni | TH | Taylor, Herman | PM | Piper, Megan | SS | Srivastava, Satish |
| TB | Thompson, B | JI | Jaspers, Ilona | CM | Caligiuri, Michael | KS | Kinlay, Scott |
| HB | Halpern-Felsher, Bonnie | SI | Stepanov, Irina | FM | Fiore, Michael | MS | Murphy, Sharon |
| RB | Rounsaville, Bruce | WI | Wainer, Irving | MM | Matthay, Michael | SS | Sharma, Sherven |
| LC | Latkin, Carl | BJ | Blalock, J | WM | Weaver, Michael | SS | Sigmon, Stacey |
| CC | Calfee, Carolyn | FJ | Frank, James | ZM | Zou, Ming-Hui | GS | Glantz, Stanton |
| LC | Lerman, Caryn | CJ | Chriqui, Jamie | WM | Wang, Mingyao | LS | London, Stephanie |
| MC | Murry, Charles | PJ | Pittet, Jean-FranÃ§ois | SM | Siahpush, Mohammad | OS | O'Malley, Stephanie |
| PC | Perry, Cheryl | FJ | Forster, Jean | BN | Benowitz, Neal | HS | Hecht, Stephen |
| CC | Chou, Chih-Ping | SJ | Smith, Jennifer | FN | Freedman, Neal | HS | Higgins, Stephen |
| AC | Abnet, Christian | UJ | Unger, Jennifer | HN | Hackett, Neil | NS | Nelsen, Stephen |
| DC | Doerschuk, Claire | FJ | Freudenheim, Jo | BN | Brewer, Noel | CS | Carmella, Steven |
| SD | Stein, Dan | MJ | Morris, John | BO | Barski, Oleg | DS | Dubinett, Steven |
| CD | Conklin, Daniel | RJ | Richie, John | DP | Derosse, Pamela | ZS | Ziegler, Steven |
| LD | Langleben, Daniel | SJ | Samet, Jonathan | LP | Ling, Pamela | KS | Krishnan-Sarin, Suchitra |
| RD | Romer, Daniel | CJ | Carroll, Joseph | RP | Reiter, Paul | JS | Jordt, Sven-Eric |
| ND | Nguyen, Dao | GJ | Guydish, Joseph | GP | Ganz, Peter | ET | Eissenberg, Thomas |
| HD | Hoon, Dave | MJ | Muscat, Joshua | SP | Shields, Peter | VT | Valente, Thomas |
| ED | Elashoff, David | CK | Cummings, K | VP | Villalta, Peter | BT | Baker, Timothy |
| SD | Schrump, David | BK | Burdick, Katherine | SP | Szeszko, Philip | LT | Lencz, Todd |
| WD | Williams, David | CK | Carroll, Kathleen | TP | Taylor, Philip | PW | Pickworth, Wallace |
| WD | Wong, David | LK | Liu, Kathleen | UP | Upadhyaya, Pramod | BW | Bailey, William |
| DD | Drayna, Dennis | JK | Jozwiak, Krzysztof | ZQ | Zhang, Qing-Yu | FX | Fang, Xiaohui |
| HD | Hatsukami, Dorothy | RK | Ribisl, Kurt | TR | Tyndale, Rachel | DX | Ding, Xinxin |
| BD | Bell, Douglas | CL | Chassin, Laurie | MR | McConnell, Rob | MY | Mineur, Yann |
| SE | Stein, Elliot | WL | Ware, Lorraine | BR | Balster, Robert | YY | Yang, Yihong |
| ME | Mongodin, Emmanuel | GL | Gerald, Lynn | TR | Tarran, Robert | WY | Wu, Yong |
| XZ | | | | Xie, Zhonglin | | | |
